# Immersive sensory evaluation: Practical use of virtual reality sensory booth

**DOI:** 10.1016/j.mex.2024.102631

**Published:** 2024-02-23

**Authors:** Abdul Hannan Bin Zulkarnain, Zoltán Kókai, Attila Gere

**Affiliations:** Department of Postharvest, Supply Chain, Commerce and Sensory Science, Institute of Food Science and Technology, Hungarian University of Agriculture and Life Sciences, H-1118 Budapest, Villányi út 29-31, Hungary

**Keywords:** Food science, Sensory analysis, Unity, Just-about-right (JAR), Check-all-that-apply (CATA), VR Sensory Booth

## Abstract

Sensory booths enhanced with VR technology have displayed promising potential for improving sensory evaluation, perception research, and educational experiences. However, there remains an insufficient of data on VR's utilization in sensory science. In our research, we designed a virtual sensory booth (SB) utilizing Virtual Reality (VR) to complement sensory analysis and foster applications in the field of sensory science. The experiment involved the utilization of diverse sensory methods and product samples for examination within the virtual SB, which was compared to the traditional SB. A total of forty-three participants took part in the study to scrutinize the implications of the virtual SB. The results of a post-VR questionnaire demonstrated the participants' positive reception of the virtual SB. The study's findings suggest that the virtual SB could serve as a valuable resource for sensory scientists and individuals keen on exploring the emerging opportunities offered by VR. Notably, the virtual SB has proven to have potential applications, particularly within the food industry, with a special focus on sensory science.

• Virtualized SB incorporating VR technology is a promising sensory evaluation and perception studies approach.
• Virtual SB intends to use various sensory methods in VR applications for sensory analysis.
• The creation of new VR-based technological solutions for sensory analysis can serve as a supplement to traditional sensory analysis.

## Graphical abstract

**Figure:**
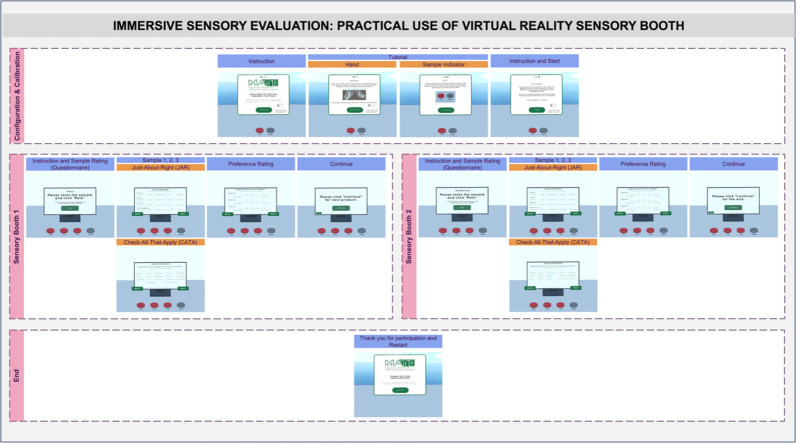

Specifications table

**Table:** 

Subject area:	Food Science
More specific subject area:	Sensory Science
Name of your method:	VR Sensory Booth
Name and reference of original method:	N.A.
Resource availability:	Suggested Device: Oculus Quest 2 Software Needed: Unity 2022.3.10f1 Plugin Needed: OVRBuild APK (optional), OpenXR Plugin 1.8.2, Oculus Integration 57.0, Oculus SDK 1.3.2 Software repository: https://github.com/MATESensoryVR/VRSensoryBooth_V1.2023.git Developer documentation/manual: https://github.com/MATESensoryVR/VRSensoryBooth_V1.2023/blob/main/README.md

**Method details**

## Motivation and significance

Virtual reality (VR) represents a paradigm shift in sensory evaluation. These immersive environments are redefining the way we analyze and understand the sensory attributes of products across various industries [1,2]. Virtualized sensory booths provide an immersive environment that enhances engagement and ecological validity compared to traditional sensory booths [3]. Research has shown that sensory evaluation conducted in immersive VR environments can result in better engagement and perception of food products [4]. The use of VR in the evaluation of alcoholic beverages has also been explored, with studies investigating the influence of environmental immersion on hedonics, perceived appropriateness, and willingness to pay [5]. Similarly, VR has been used in wine tasting experiments to assess the effects of context and virtual reality environments on the tasting experience, acceptability, and emotional responses of consumers [6].

Immersive VR environments have also been studied in the context of sensory perception of beef steaks and chocolate [7]. These studies aim to simulate different eating environments and understand how consumers' sensory responses to food are influenced by virtual reality. Additionally, the comparison of sensory engagement in traditional sensory booths and alternative environments, such as study commons, has been explored in the evaluation of tea and cola [8].

The potential applications of VR and augmented reality (AR) technologies in sensory science are vast. These technologies offer new possibilities for collecting and processing sensory and consumer information [9,10]. Furthermore, VR has been employed in educational settings, enhancing learning experiences and providing immersive environments for students [11]. The use of VR in sensory science and perception research has opened up new avenues for studying sensory immersion and its impact on perception and engagement. These studies have demonstrated the potential of VR technology to create realistic and immersive sensory experiences, allowing researchers to investigate the influence of various factors on sensory perception [12,13].

In this work, we present the virtual Sensory Booth (SB), which serves as a basis and support for the development of VR applications focused on different methods of sensory analysis on various product samples. The proposed software offers fundamental functions for creating a virtual SB. The software is a further development from ongoing research that can be found in [14], which is more focused on the sensory laboratory, and the new version of the application has been developed further by using a different software developer with a focus on sensory booths. The software description and functionalities are described in Section 2 with the architectural scenes. An illustrative example is explained in Section 3, and the impact of the application is discussed in Section 4. Lastly, further development and the conclusion are presented in Section 5.

## Software procedure and description

First, download Unity version 2022.3.10f1 (Unity Technologies, Unity Software Inc., San Francisco, California, US). Access the software by deploying the asset via the provided link: https://github.com/MATESensoryVR/VRSensoryBooth_V1.2023.git. Next, launch the software with Unity. From there, you can enjoy the software on VR devices, such as Oculus Quest 2, or tailor it to suit your preferences. The link to the developer documentation/manual can be found at https://github.com/MATESensoryVR/VRSensoryBooth_V1.2023/blob/main/README.md.

**Fig. 1:**
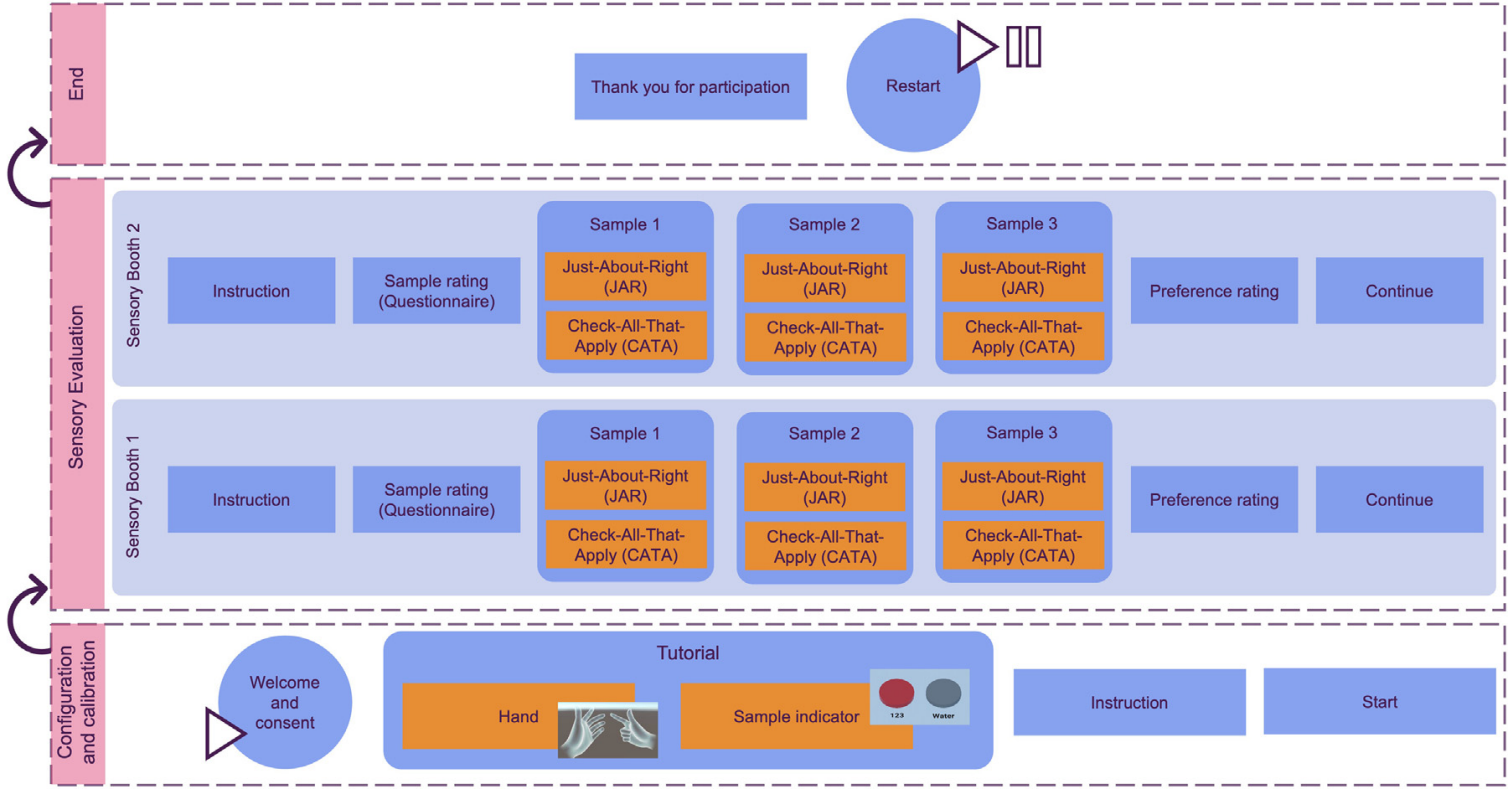
Multi-layer scenes architecture for the development of virtual sensory booth (SB) application. The application consists of three main layers: (i) configuration and calibration (introductory), (ii) sensory evaluation (SB 1 and 2) and (iii) end scene.

Fig. 1 illustrates a multilayer scene developed for sensory evaluation. The software was developed and designed using Unity version 2022.3.10f1 (Unity Technologies, Unity Software Inc., San Francisco, California, US) and C++ for Oculus Quest 2 (Reality Labs, Meta Platforms Inc., Menlo Park, California, US). The VR sensory booth was designed to closely resemble the sensory booth at the Hungarian University of Agriculture and Life Sciences (MATE). Following the ISO 8589:2007 standard [15] guidelines, a well-established sensory laboratory must use white (or light grey) colors, good natural lighting (6500 K), and well-ventilated air.

In the virtual SB, there is a setup that includes a computer, monitor, chair, and a sample indicator with three (3) randomized digits. Additionally, there is water with the booth's dimensions measuring 1 m x 1 m x 2.5 m.

The layered scenes provide instructions and steps for sensory evaluation, focusing on blind test functionality. This sensory test is limited to the Just-about-right (JAR) and Check-all-that-apply (CATA) sensory methods. For further details on the incorporated sensory methods, see Ares and Jaeger work [16]. The application is divided into three main layers: (i) configuration and calibration (introductory), (ii) sensory evaluation, and (iii) the end scene.

### Scene functionality

#### Configuration and calibration (introductory) layer

First, the configuration and calibration scene plays a crucial role in ensuring that the virtual sensory booth is calibrated to meet the specific requirements of the participants, such as adjusting the height, setting the distance of the sample, ensuring the clarity of the scene, and accommodating participants wearing eyeglasses. Calibration is only required once per participant. This scene provides clear instructions and tutorials for the tasks. It also initiates the hand interaction tutorial, which is essential for enabling participants to interact with the virtual SB in a meaningful and engaging way.

**Fig. 2:**
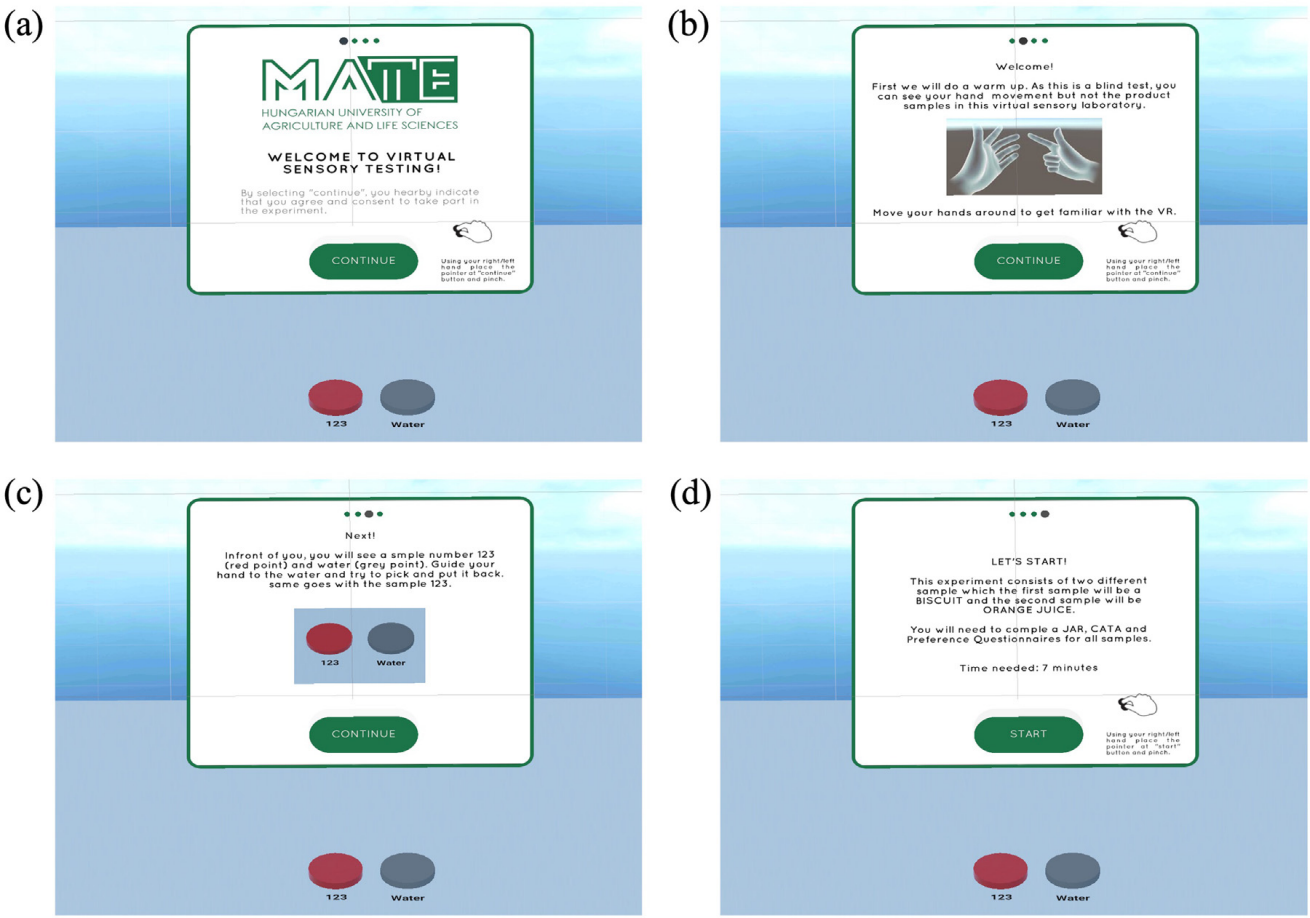
Configuration and calibration (introductory) scene steps; (a) Welcome note and consent, (b) Tutorial on hand tracking, (c) Tutorial on sample indicator, (d) Sensory instruction on methods and products, and starting point.

Based on Fig. 2, the scene comprises several steps. Step 1 [Fig. 2(a)] involves displaying a welcome note and obtaining consent from participants to ensure they are aware of the study's objectives. Participants can proceed by clicking the 'Continue' button. The subsequent steps are part of a tutorial, designed as a warm-up session, especially for participants who are new to VR. Step 2 [Fig. 2(b)] focuses on hand tracking, allowing participants to use their own hands with the guidance of animated hands showing them how to interact with the VR environment, as the Quest 2 VR headset requires a pinching motion (using the index finger and thumb) for clicking. Step 3 [Fig. 2(c)] introduces the sample indicator, where participants can practice picking up and putting back food samples (in this experiment, chocolate biscuits and orange juice). This step also serves as a calibration process for the laboratory assistant to ensure the correct placement of the samples on the right indicator. The final step, step 4 [Fig. 2(d)], displays an instruction page specifying product sample categories, sensory evaluation methods, and the estimated time required for the entire testing process. By clicking 'Start,' the next scene will appear. It's worth noting that all the instructions, images, and product samples can be customized in the Unity software.

#### Sensory evaluation layer

Secondly, the sensory evaluation layer serves as the core of the application and is responsible for conducting sensory testing on two types of products, as well as answering the sensory questionnaire. In this application, both just-about-right (JAR) and check-all-that-apply (CATA) tests are provided for each sample, allowing participants to engage with the virtual SB.

**Fig. 3:**
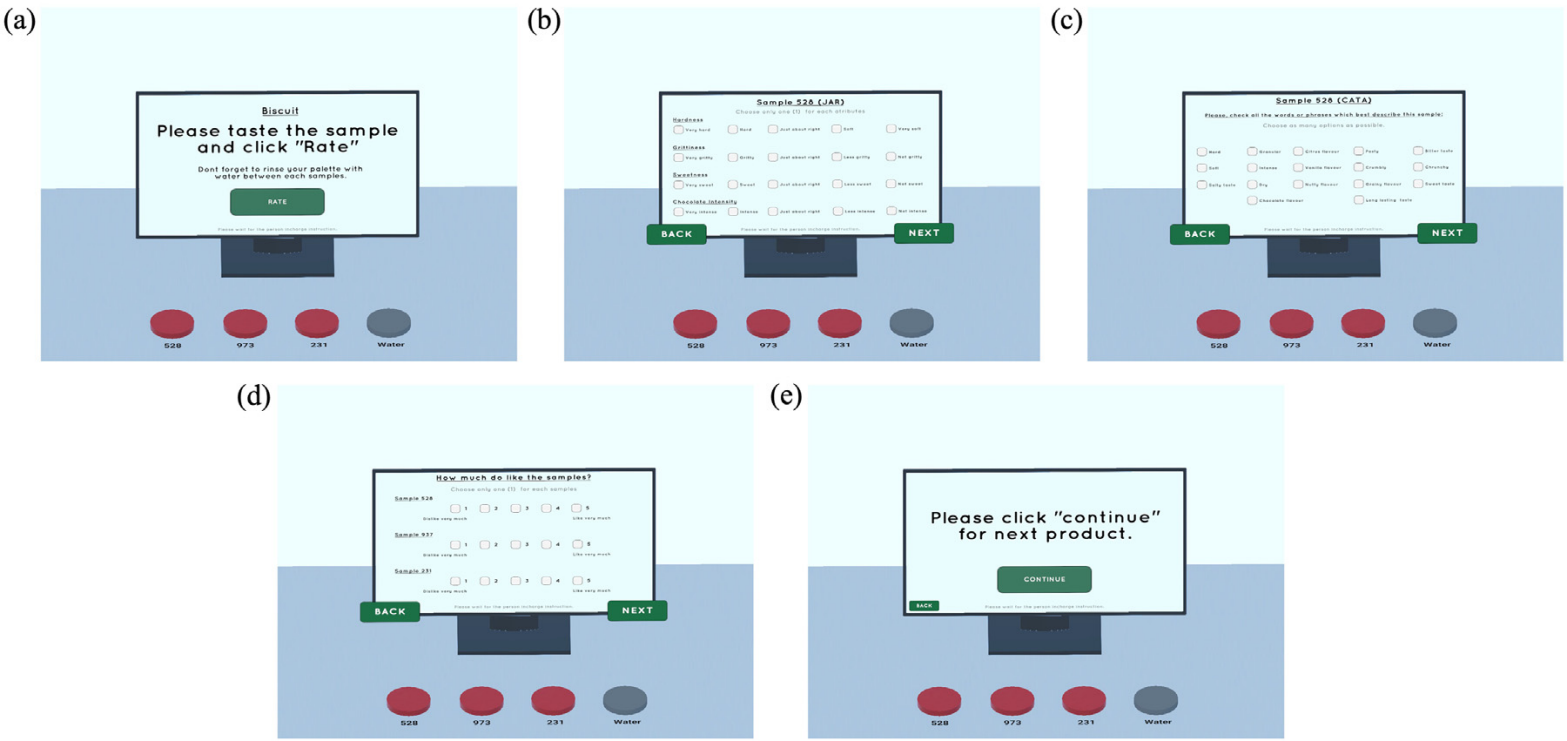
Sensory evaluation booth 1 scene steps; (a) Instruction page with product sample, (b) JAR for samples (will be repeated 3 times with 3 different samples), (c) CATA for samples (will be repeated 3 times with 3 samples), (d) Preference on each sample, (e) Finish evaluation for product sample and continue to next product.

Fig. 3 displays the step scenes for the products. Both SB 1 and 2 have the same flow; the only difference lies in the product sample and its attributes. In both SB 1 and 2, step 1 [Fig. 3(a)] presents an instruction page regarding the type of product, and by pressing the 'Rate' button, participants proceed to the next steps. On the table, random three-digit numbers indicate different product samples for testing. Steps 2 [Fig. 3(b)] and 3 [Fig. 3(c)] in both scenes for SB 1 and 2 are repeated alternately, with the JAR questionnaire coming first, followed by the CATA questionnaire, and this cycle is performed three times for each sample number indicated on the table. Step 4 [Fig. 3(d)] involves rating the preference and liking of each sample using a 5-scale (Likert scale) to determine the preferred product. Finally, step 5 [Fig. 3] serves as an indicator that the product sensory test is complete, and participants can continue to the next product or the end scene.

All the instructions, images, product samples, sample numbers, and questionnaire attributes can be changed within the Unity software.

#### End layer

**Fig. 4:**
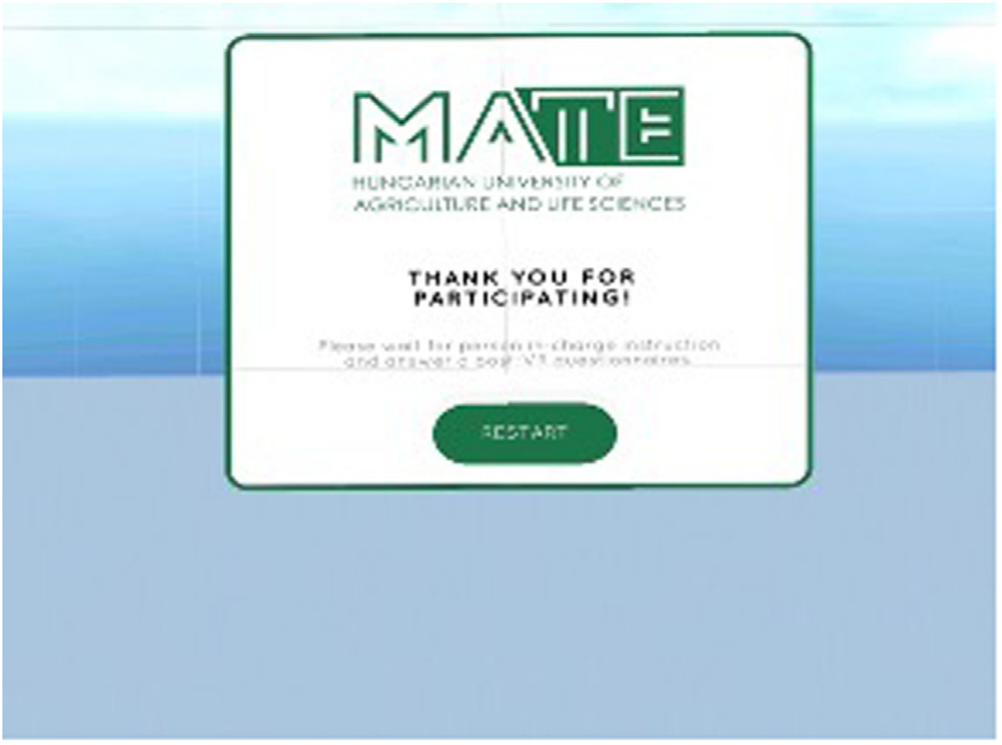
End scene with a restart button.

Finally, the end scene (Fig. 4) indicates to participants that the experiment is finished and it can be restarted for the next participant.

In the end scene, participants are thanked for participating in the test. The restart button can be clicked by the laboratory assistant to repeat the process for the next participant. Instructions and images can be changed within the Unity software.

### Method validation

To evaluate the usability of the application, the post-VR questionnaire is used to investigate the acceptability of a virtual SB using VR. The evaluation process involved 43 respondents from MATE, aged between 20 and 40 years (mean±SD: 25.16 ± 3.98). Of these participants, 48.84% had no prior experience with VR, while 51.16% had previous experience with VR technology.

**Fig. 5:**
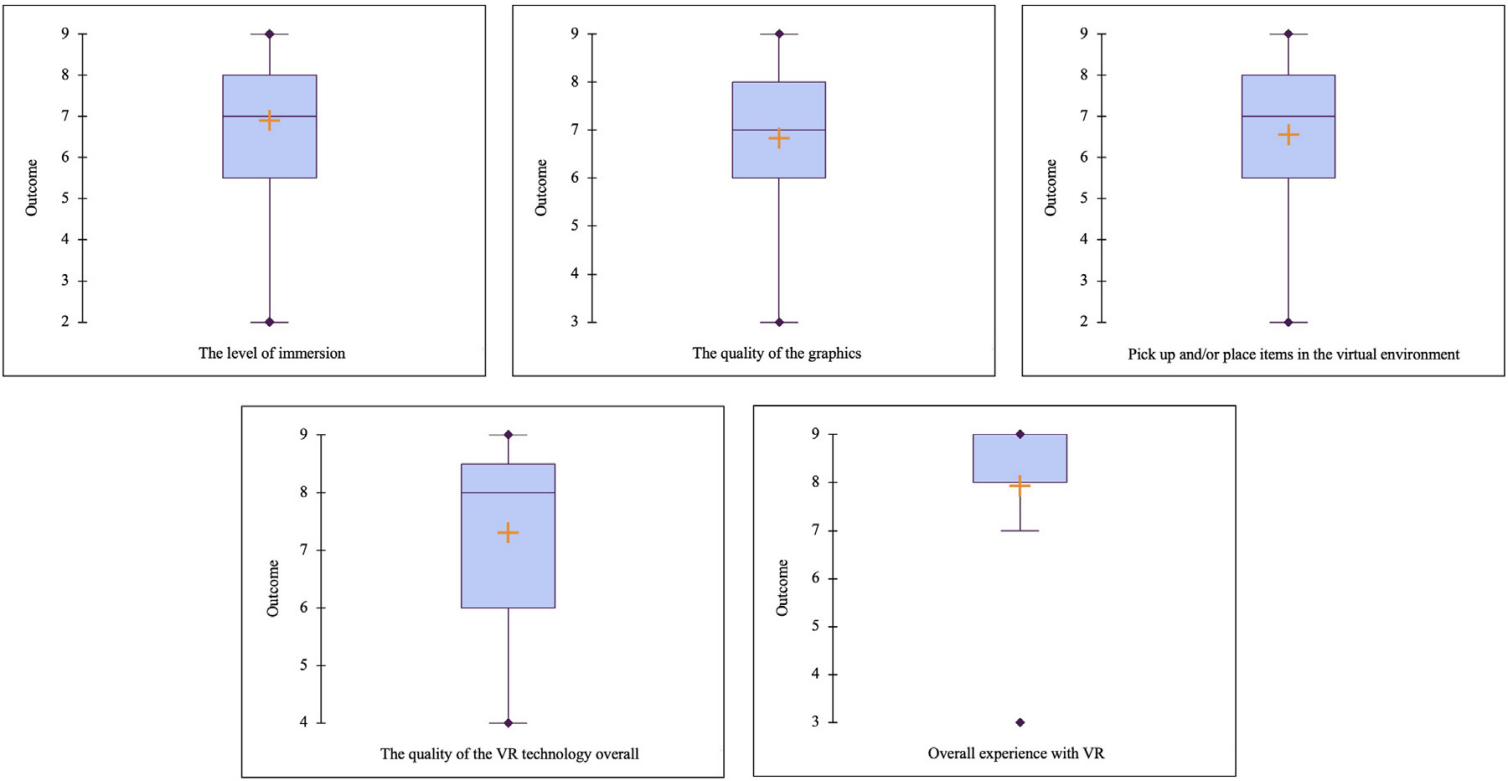
Box plot of each post-VR questionnaire results.

Fig. 5 displays a box plot of each post-VR questionnaire results. The post-VR questionnaire serves the purpose of measuring the immersive level and the acceptability of the VR SB. Some parts of the post-VR questionnaires were adapted from Virtual Reality Neuroscience Questionnaire (VRNQ) [17]. It comprises five questions, addressing the level of immersion, the quality of graphics, the ability to pick up and place items in the virtual environment, the overall quality of the VR technology, and the overall experience with VR. Participants provide ratings for the virtual SB by selecting a value on a parameter scale between 1 (very low/very difficult/negative) and 9 (very high/very easy/positive), with higher values indicating a more favorable experience (Likert Scale).

The findings were statistically interpreted and presented in tabular and graphical form, including the mean or average value, minimum, maximum, and standard deviation. Descriptive analysis was performed on the post-VR questionnaire scores using XLSTAT (Addinsoft, New York, USA). Table 1 presents the statistical results for each post-VR questionnaire.

**Table 1:** Statistical results of each post-VR questionnaire.

Post-VR questions	Score		Average		
	Minimum	Maximum	Mean	±	Std Dev.
The level of immersion.	2	9	6.91	±	1.67
The quality of the graphics.	3	9	6.84	±	1.66
Pick up and/or place items in the virtual environment.	2	9	6.56	±	2.02
The quality of the VR technology overall.	4	9	7.30	±	1.44
Overall experience with VR.	3	9	7.93	±	1.28

All of the scores for the post-VR questionnaire are above 6. The 'Overall experience with VR' received the highest average score (7.93 ± 1.28), while the lowest was 'Pick up and/or place items in the virtual environment' (6.56 ± 2.02). The scores for the other questions, in descending order, are as follows: 'The quality of the VR technology overall' (7.30 ± 1.28), 'The level of immersion' (6.91 ± 1.67), and 'The quality of the graphics' (6.84 ± 1.66). This indicates that the virtual SB was well-received, and participants found the experience to be immersive when using VR.

## Illustrative example

To help readers visualize what the virtual SB offers, we have created a complementary video (https://youtu.be/XsZzttValG8) that provides a participant's perspective of the virtual world they would experience during sensory evaluation. This video demonstrates the steps in the scenes and the sensory analysis process.

**Fig. 6:**
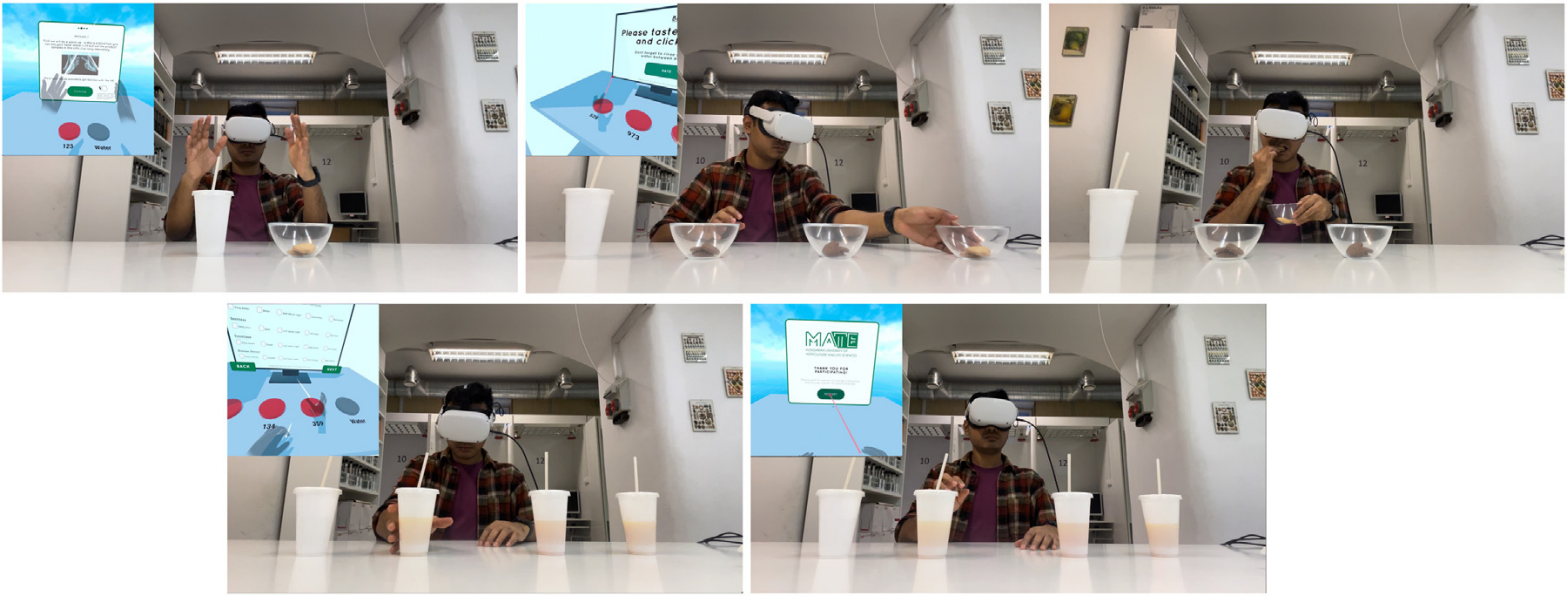
Point of view of participant and real time game play VR mode sensory analysis with Oculus Quest 2 VR headset.

The virtual SB application, developed in Unity, is deployed in VR mode. This mode encompasses the content and environment necessary for the experiment. Fig. 3 illustrates how users can utilize both modes. Fig. 6 displays the VR mode using an Oculus Quest 2 device, providing the view from the participant's perspective.

## Impact

We developed the Virtual SB using the Unity game engine and integrated it with the Oculus Integration SDK. This SDK enables the application to be deployed on any Oculus Virtual Reality headset, specifically the Oculus Quest 2. To enhance the application's immersion, we implemented integration and hand tracking. We used the OpenXR Plugin and Oculus Integration for hand tracking, allowing participants to touch samples with their real hands instead of a controller. This significantly boosts the immersive experience for the participants, aiming to make it as close to a real sensory booth as possible.

Moreover, we designed the virtual SB in VR mode, which offers potential benefits for researchers and sensory scientists. This technology provides broader accessibility, allowing it to be accessed from anywhere and at any time. The virtual SB can be used to showcase digital assets in an immersive environment. Additionally, it can be adapted to simulate various real-world environments, such as restaurants, cafes, parks, and more. This adaptability provides researchers and sensory scientists with access to environments that might otherwise be inaccessible or expensive to recreate. Our virtual SB offers a ground-breaking approach to investigating the potential of VR within an ever-evolving scientific landscape, focusing on context. VR holds significant potential for the development of practical and advanced immersive applications within the food industry.

## CRediT authorship contribution statement

**Abdul Hannan Bin Zulkarnain:** Conceptualization, Methodology, Software, Formal analysis, Investigation, Writing – original draft, Writing – review & editing, Visualization. **Zoltán Kókai:** Supervision, Writing – original draft, Writing – review & editing. **Attila Gere:** Conceptualization, Methodology, Validation, Writing – original draft, Writing – review & editing, Supervision.

## Declaration of competing interests

The authors declare that they have no known competing financial interests or personal relationships that could have appeared to influence the work reported in this paper.

## Data Availability

Data will be made available on request.
